# Analysis of the current risk of *Leishmania infantum* transmission for domestic dogs in Spain and Portugal and its future projection in climate change scenarios

**DOI:** 10.3389/fvets.2024.1399772

**Published:** 2024-05-02

**Authors:** Iván Rodríguez-Escolar, Alfonso Balmori-de la Puente, Manuel Collado-Cuadrado, Daniel Bravo-Barriga, Sarah Delacour-Estrella, Ricardo Enrique Hernández-Lambraño, José Ángel Sánchez Agudo, Rodrigo Morchón

**Affiliations:** ^1^Zoonotic Diseases and One Health GIR, Biomedical Research Institute of Salamanca (IBSAL), Faculty of Pharmacy, University of Salamanca, Salamanca, Spain; ^2^Departamento de Salud Animal, Grupo de Investigación en Salud Animal y Zoonosis (GISAZ), Facultad de Veterinaria, Universidad de Córdoba, Córdoba, Spain; ^3^Instituto Agroalimentario de Aragón, Departamento de Patología Animal, Facultad de Veterinaria, Universidad de Zaragoza, Zaragoza, Spain; ^4^Research Group on Biodiversity, Human Diversity and Conservation Biology, University of Salamanca, Salamanca, Spain; ^5^Centre for Environmental Studies and Rural Dynamization (CEADIR), University of Salamanca, Salamanca, Spain

**Keywords:** *Leishmania infantum*, leishmaniosis, *Phlebotomus perniciosus*, Spain, Portugal, dogs, ecological niche model, infection risk

## Abstract

Canine leishmaniosis, caused by the protozoan parasite *Leishmania infantum*, is a cosmopolitan vector-borne zoonosis, transmitted principally by *Phlebotomus perniciosus* in Spain and Portugal, where it is considered an endemic disease. Ecoinformatics tools such as ecological niche models (ENM) have been successfully tested to model the distribution of the risk of infection of different parasitosis as they take into account environmental variables vital for their survival. The risk map proposed in this study combines the potential distribution of *Ph. perniciosus* in the Iberian Peninsula and the calculation of the infection rate of the parasite in the vector to model the risk of contracting the disease in a more realistic way. In fact, this weighting strategy improves the predictive power of the resulting model (*R*^2^ = 0.42, *p* = < 0.01) compared to the *Ph. perniciosus* ENM model alone (*R*^2^ = 0.13, *p* > 0.05). The places with the highest risk of transmission are the southwest and central peninsular area, as well as the Mediterranean coast, the Balearic Islands and the Ebro basin, places where the ideal habitat of *Ph. perniciosus* and the infection rate is also high. In the case of future projections under climate change scenarios, an increase in the risk of infection by *L. infantum* can be observed in most of the territory (4.5% in 2040, 71.6% in 2060 and 63% in 2080), mainly in the northern part of the peninsula. The use of ENMs and their weighting with the infection rate in *Ph. perniciosus* is a useful tool in predicting the risk of infection for *L. infantum* in dogs for a given area. In this way, a more complete model can be obtained to facilitate prevention and control.

## 1 Introduction

Vector-borne zoonotic diseases pose significant health challenges for both animals and humans, accounting for 61% of human diseases of zoonotic origin (1–4). These diseases are increasingly prevalent across the European continent due to globalization and climate change. Factors such as rising temperatures, vector movement, increased migration and tourism involving infected people and animals, and inadequate management diseases control measure among other factors, contribute to this trend (5–8).

Canine leishmaniosis stands as a vector-borne zoonotic disease caused by *Leishmania infantum*, a protozoan parasite that affecting both animals and humans alike, with dogs being the main domestic reservoir. Its primary vectors in Iberian Peninsula are *Phlebotomus perniciosus* and *Phlebotomus ariasi* species (9, 10). These, when feeding on blood from the definitive host, ingest amastigotes (tissue form), which then develop to promastigotes (infective form) in the intestine of the vector and subsequently migrate to the proboscis (11). This process is temperature-dependent, increasing logarithmically the percentage of sandflies infected by *L. infantum* between 10 and 30°C, the survival range of the vector (12).

Its distribution is cosmopolitan and dynamic, both spatially and temporally, subject to multiple social and environmental factors. In Europe, the countries located in the Mediterranean basin (France, Greece, Italy, Spain, and Portugal) are endemic, with a much higher incidence of canine leishmaniosis than human leishmaniosis (13). Within the entire peninsular and insular territory of Spain and Portugal, most of its surface is considered endemic. In Spain, reported seroprevalences range from 0.86 to 24.66%, with the highest reports in the south and on the Mediterranean coast (14–16). In Portugal, canine leishmaniosis is found throughout the territory with a heterogeneous distribution, with the highest seroprevalence observed in the center of the country, with values close to 30% (13).

In the context of prevention and control of animal and human leishmaniosis, it is essential to emphasize the various tools used for the prevention of infection (17). One of these is mapping to visualize areas where there is a risk of disease infection, as it allows early identification of risk areas, facilitates planning of interventions, optimizes resource allocation, supports epidemiological surveillance and improves risk communication to the population. Ecoinformatics tools, such as Geographic Information Systems (GIS) and ecological niche models (ENM), can be employed to manage zoonotis parasitosis. These tools facilitate modeling the distribution of the disease by considering the bioclimatic and environmental variables necessary for their maintenance (18). ENMs assign suitability values to the environmental habitats where an organism lives, achieved through the correlation between the known distribution records of the species and the environmental variables that influence it (19). These models have already been used to assess the potential risk of zoonotic disease transmission utilizing records of parasite presence, infected hosts (20, 21) and potential transmitting vectors (22, 23).

For leishmaniosis, these tools have been specifically applied specifically to the Mediterranean basin and other parts of the world to model the risk of infection concerning environmental variables such as precipitation, temperature, and vegetation (17, 24–32). Local studies in the Iberian Peninsula have assessed the risk of L*. infantum* infection using GIS tools. The initial risk map was constructed in the community of Madrid based on the distribution of vectors (*Ph. perniciosus* and *Ph. ariasi*), indicating high risk nuclei in individualized foci in the Center and South of the region (33). The second study, in East-Central Portugal, was focused solely on the presence of infected hosts, and suggests that irrigated crops and olive groves, open forests, and watercourses influence infection distribution (34). However, these studies did not integrate ecological niche models of the vectors with parasite development within them, extracting the full potential of these techniques and being much more realistic. The ability to model the development of the parasite inside the vectors together with the distribution of the latter through ENMs, has made infection risk mapping a supplementary tool in control plans for other vector-borne diseases, such as dirofilariosis on a larger scale (35–37).

The aim of this study was to develop an infection risk map for *L. infantum* in the Iberian Peninsula (Spain and Portugal) and the Balearic Islands as well as its projection to 2080 through the use of ENM, taking into account the habitat suitability of *Ph. perniciosus*, its main vector in the study area, and the calculation of the infection rate of *L. infantum* in the vector.

## 2 Materials and methods

### 2.1 Area of study

**Figure 1:**
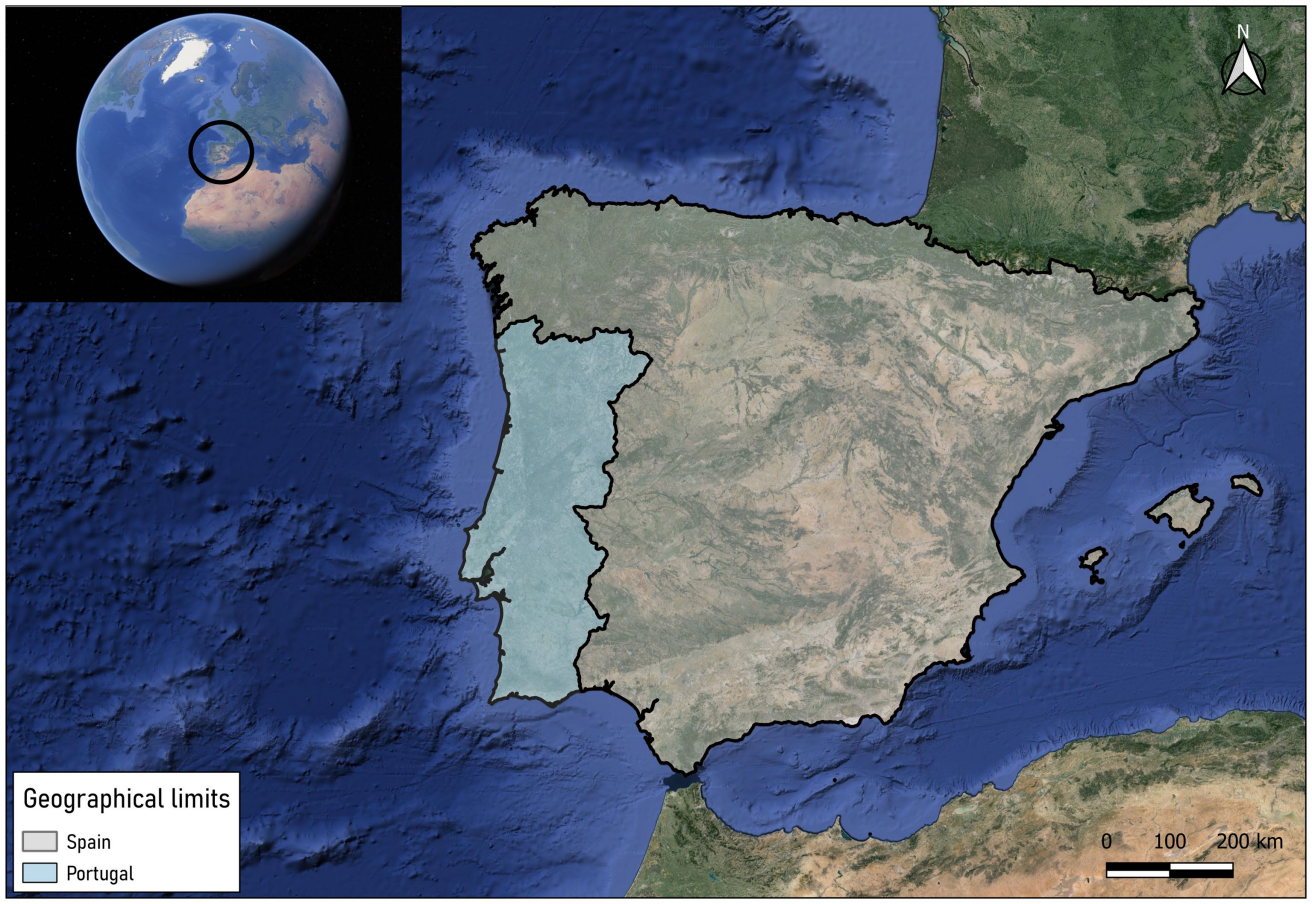
Location of Portugal and Spain and Balearic Islands (Spain).

The Iberian Peninsula (40°14′24” N 4°14′21” W), formed by the countries of Spain and Portugal, and the Balearic Islands (Spain), were established as a study area. This territory is located in the southeast of the European continent, close to Morocco (Africa) and only separated from it by the Strait of Gibraltar (Figure 1). Both Spain and Portugal have overseas territories such as the Canary Islands, the Azores or Madeira, but these have not been taken into account in this study due to the particularity of their biogeographical characteristics, which are very different from those of the continent. The Iberian Peninsula covers a territory of approximately 590,000 km^2^ and the Balearic Islands 4,992 km^2^. Most of the peninsular territory is surrounded by coastline, surrounded to the east and south by the Mediterranean Sea, to the north by the Cantabrian Sea and to the west by the Atlantic Ocean. Regarding the continental territory, it is mostly made up of a large plateau with an average altitude of 600 meters crossed by both large hydrographic basins and a multitude of mountain ranges, providing the peninsula with a great diversity of environments. The mountain ranges that divide the peninsula are the Pyrenees, the Cantabrian Mountains, the Iberian System, the Central System and the Penibaetic System. The basins of the Ebro River (northeast) and the Guadalquivir River (south) are the main river basins of the peninsula, followed by other smaller basins such as the Guadiana (southwest), Júcar and Segura (east), Duero and Tajo (west) and Miño (northwest).

The Iberian Peninsula has a wide variety of climates, which give it a great biological importance. The northwest of the peninsula is an area of cool summers, mild winters and high humidity and precipitation throughout the year. The Levantine coast is an area with a Mediterranean climate with hot, dry summers and mild winters. The south is characterized by a warm and dry African-influenced climate with summer drought; while, in the central plateau, whipped by strong winds, the climate is very hot in summer and very cold in winter with rainfall normally restricted to spring and autumn. It is worth noting the notable difference in the climate with respect to the altitude, with the high mountain areas having mild summers and being covered with snow in winter (38).

### 2.2 *Phlebotomus perniciosus* habitat suitability modeling

#### 2.2.1 Distribution data

To model habitat suitability for *Ph. perniciosus*, the main vector of canine leishmaniosis in the Iberian Peninsula and Europe (14, 39–41), we collected records of presence of this species from published studies (9, 42–44) and Global Biodiversity information Facility (GBIF) (45) to obtain the most representative face-to-face attendance possible. In order to obtain only presence points and eliminate sampling biases, the data were processed at a resolution of 1 km^2^. Finally, 3,032 vector presence data were obtained for use in the model.

#### 2.2.2 Bioclimatic and environmental data

At a spatial resolution of 1 km^2^, 19 bioclimatic variables related to temperature and precipitation were downloaded from World Clim (46), for present-day conditions and projected scenarios for 2040, 2060, and 2080 (47). Subsequently, a multicollinearity analysis was performed on these variables in the R software using Pearson’s correlation coefficient (48). To avoid cross-correlation between the 19 bioclimatic variables, those with a value of *r* > ±0.75 were eliminated and taking into account the biological needs of the vector, the variables chosen were BIO_1_ (Annual Mean Temperature), BIO_2_ (Mean Diurnal Range: The mean of the monthly temperature ranges), BIO_3_ (Isothermality: Mean Diurnal Range (BIO_2_) / Temperature Annual Range (BIO_7_) × 100), BIO_8_ (Mean Temperature of Wettest Quarter), BIO_12_ (Annual Precipitation) and BIO_15_ (Precipitation Seasonality: Standard deviation of weekly or monthly precipitation values as a percentage of the mean of those values). Next, the environmental variables were downloaded: density of shrubs and herbaceous plants (49) and the human footprint (50) which includes 8 variables (built environment, population density, electric power infrastructure, farmland, grazing land, roads, railways and waterways) reflecting the impact of human activities on the ecosystem. All downloaded data layers were processed in ArcMap 10.8 to ensure uniform extent, resolution (1 km^2^ per pixel) and coordinates system (GCS_WGS_1984).

#### 2.2.3 Modeling approaches

The maximum entropy algorithm MaxEnt was used (51) to model the vector’s ecological niche from the Kuenm package of the R software (version 4.3.0) (48), automating the process (52). MaxEnt employs points of presence and environmental variables to estimate habitat suitability, which can be defined as the area in where specific environmental conditions necessary for the survival or reproduction of a species exist (53). To model *Ph. perniciosus*, 119 models were created with Kuenm for a set of variables, 17 regularization multiplier values (0.1–1.0 at 0.1 intervals, 2–6 at intervals of 1, 8, and 10) and the seven possible combinations of three feature classes (linear, quadratic, and product). The performance of the models created was assessed considering the significance of the partial receiver operating characteristic (partial ROC), with 100 iterations and 50% data for bootstrapping, skip rates (OR = 5%) and model complexity (Akaike information criterion - AIC). From the models that met the evaluation criteria, the final model was chosen based on the mean ratio of the area under the curve (AUC) obtained with points of occurrence independent of the calibration. The best-fit model (final model) was generated using the same parameters selected in the previous step. Ten replicates were developed per bootstrap with logistic outputs, and re-evaluated based on criteria ROC_parcial, OR and AICc.

### 2.3 *Leishmania infantum* infection rate in phlebotomine

The infection rate (% of *Ph. perniciosus* infected by *L. infantum*) was calculated as Rioux et al. (12), applied to our vector, by using the formula $y=0.718[1-e^{-0.237\left(x-8\right)}$] (y is % of *Ph. perniciosus* infected by *L. infantum* and x is the Annual Mean Temperature). The frequency distribution was adjusted to a theoretical ascending logarithmic curve, which allowed estimating the infection rate between 10 and 30°C, which is the temperature range in which the parasite can survive and replicate in the *Ph. ariasi* vector, as reported by Rioux et al. (12). The infection rate was carried out using the program R-4.3.0.

### 2.4 *Leishmania infantum* risk map and its validation

An infection risk map for *L. infantum* is a visual representation that identifies the geographical areas, within a given environment, where a certain risk of parasite transmission (high, medium or low) may exist. The risk of *L. infantum* infection refers to the probability that a host may become infected taking into account different factors such as the presence of the vector, the prevalence of the disease in a given population, the presence of natural reservoirs of the pathogen, environmental conditions favorable for vector reproduction, the availability of standing water for vector reproduction, and the proximity between vectors and hosts, among other factors. To generate the risk map of *L. infantum* infection in the study area, once the final ENM for *Ph. perniciosus* was generated, it was multiplied using a weighting approach with the map of the infection rate of *L. infantum* in *Ph. perniciosus*.

Our risk map was validated employing a regression analysis between the mean risk of infection and the seroprevalence of canine disease in all the autonomous communities of Spain and regions of Portugal, reported by Almeida et al. (13) and Montoya-Alonso et al. (16). In addition, geolocations of dogs infected by *L. infantum* were superimposed on the risk map. The geolocation of these infected animals is also derived from the same studies previously employed (13, 16). Simultaneously, seroprevalence data were compared using the unweighted vector ENM with the same approximation.

### 2.5 Forward projection and rank change analysis

Three suitable habitats for *Ph. perniciosus* were generated with the previously selected parameters, incorporating projections of the bioclimatic variables analyzed for the time periods 2021–2040 (2040), 2041–2060 (2060) and 2061–2080 (2080). The RCP 8.5 scenario, which represents high CO_2_ emissions, was utilized using the HadGEM3-GC21-LL model (54), to study the effect of climate change in the future, because of high greenhouse gas emissions in Europe (55). The infection rate of *L. infantum* corresponding to each of the three future scenarios was also calculated using the BIO_1_ (Annual Mean Temperature) of each time period.

The suitability habitats were weighted with the rate of infection of *L. infantum* and risk maps corresponding to each of the three periods analyzed were generated. Subsequently, the current risk map and the three projected future risk maps were transformed into presence/absence binary maps using the logistic threshold of training presence of the 10th percentile of the current map. This process is essential to perform a range-change analysis in order to establish alterations in the risk of *L. infantum* infection in the future. Finally, the percentage of cells that gained or lost risk of infection as a result of climate change was calculated for the maps projected to 2040, 2060, and 2080 compared to the present map using the biomod2 package of the R software (56).

## 3 Results

### 3.1 Habitat suitability model for *Phlebotomus perniciosus*

**Figure 2:**
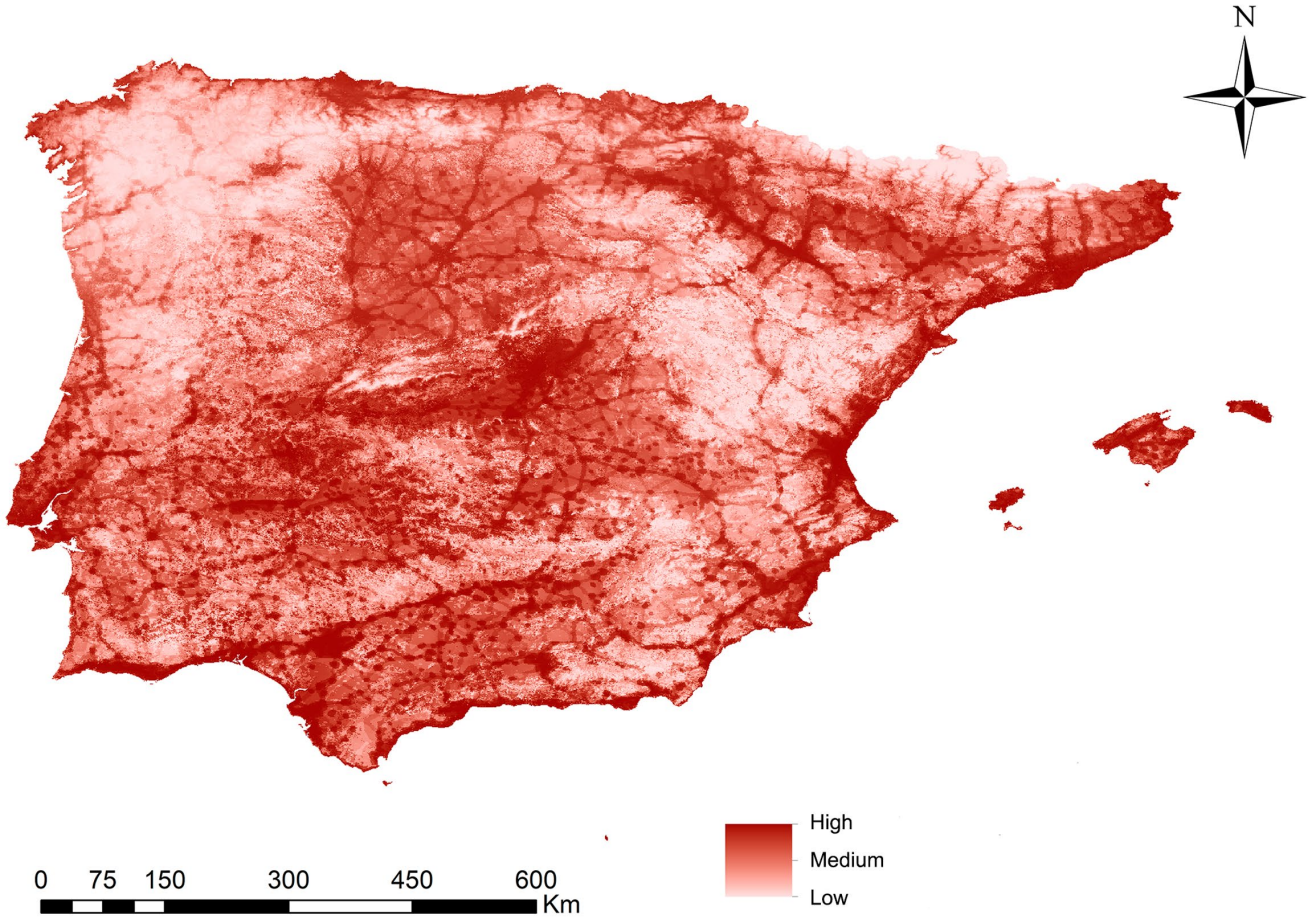
Ecological niche model (suitable habitat) for *Phlebotomus perniciosus* in Iberian Peninsula (Portugal and Spain) and Balearic Islands (Spain).

Figure 2 shows the developed ENM, indicating the suitability of *Ph. perniciosus* habitat across the Iberian Peninsula and the Balearic Islands. The maximum suitability value recorded was 0.83 (high suitability), while the minimum value was 0.0004 (low suitability). Table 1 shows the contribution degree of each of the variables in the vector model, with the variables with the highest percentage of contribution attributions to the human footprint (40.14%) and the BIO_1_ (20.83%). The remaining variables contribute between 9.37 and 2.53%, with the latter being the lowest percentage. Those areas with a high suitability value correspond to the southwest of the peninsula, followed by others such as the center, the Levantine coast and the Balearic Islands, the Ebro basin and some areas of the north coast of the peninsula. Conversely, regions in the interior northwest and the central east, as well as the mountainous areas characterized by lower population density and cooler temperatures, exhibits less suitable habitat.

**Table 1:** Analysis of the contribution of the 9 environmental and bioclimatic variables to the ecological niche model for *Phlebotomus perniciosus*.

Variable	Percent contribution
Humanfootprint	40.14%
BIO_1_ (annual mean temperatura)	20.83%
BIO_2_ (Mean Diurnal Range)	9.37%
BIO_12_ (anual precipitation)	8.93%
Shrubs density	6.63%
BIO_3_ (isothermality)	4.48%
Herbaceous density	4.36%
BIO_8_ (mean temperature of wettest quarter)	2.73%
BIO_15_ (precipitation seasonality)	2.53%

### 3.2 Map of *Leishmania infantum* infection rate in *Phlebotomus perniciosus*

**Figure 3:**
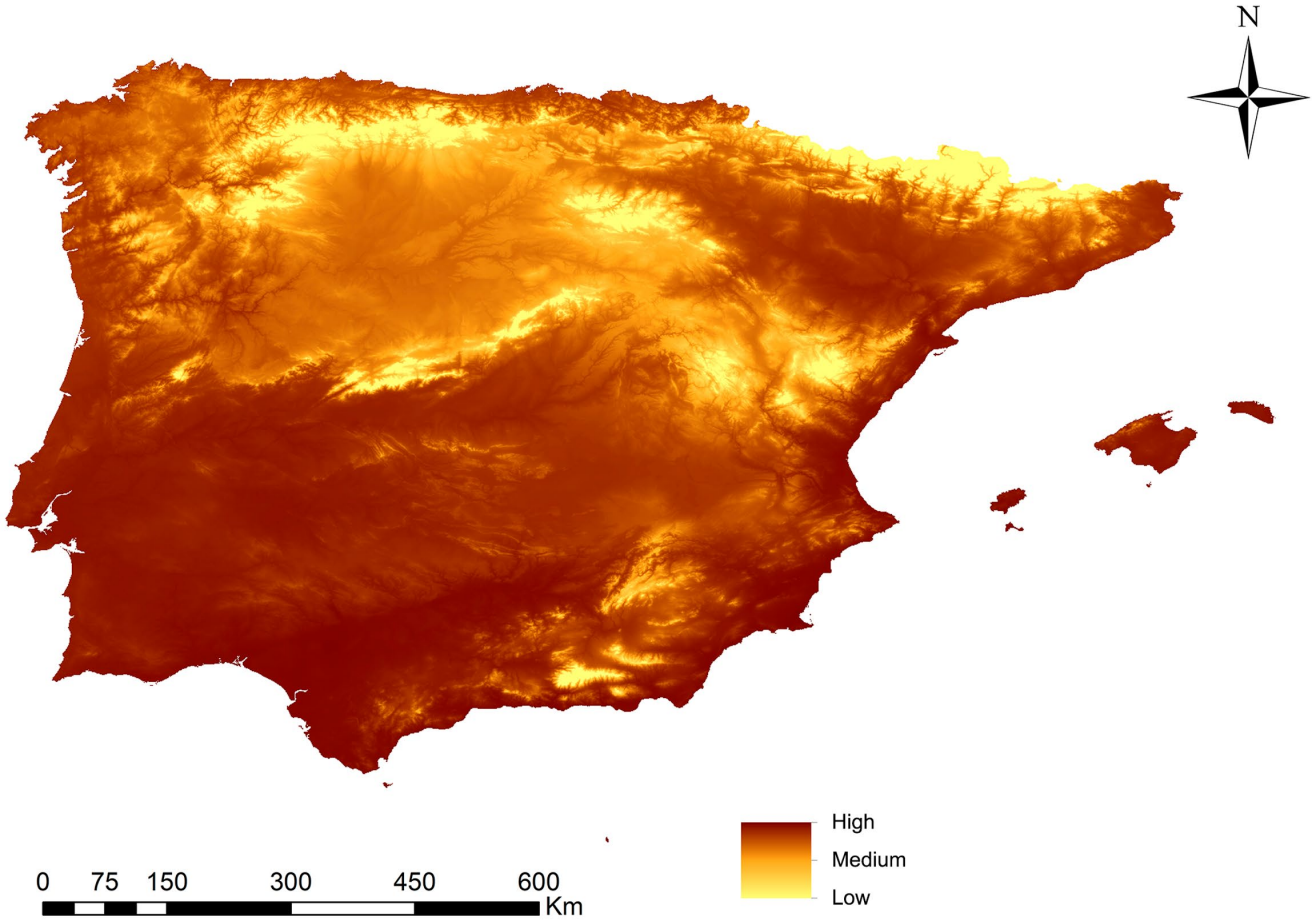
Prediction of *Phlebotomus perniciosus* infection rate in Iberian Peninsula (Portugal and Spain) and Balearic Islands (Spain).

Figure 3 illustrates the potential resulting map depicting the rate of *L. infantum* infection in *Ph. perniciosus* across the Iberian Peninsula and the Balearic Islands. Areas with the highest infection rate are concentrated to those of the southwest and south of the peninsula, the Mediterranean coast, the Balearic Islands and the Ebro basin, and the north and northwest coasts of the territory. The northern plateau had a medium infection rate, while the mountain areas, at higher altitudes display percentages close to 0.

### 3.3 Map of potential risk of transmission of *Leishmania infantum*

**Figure 4:**
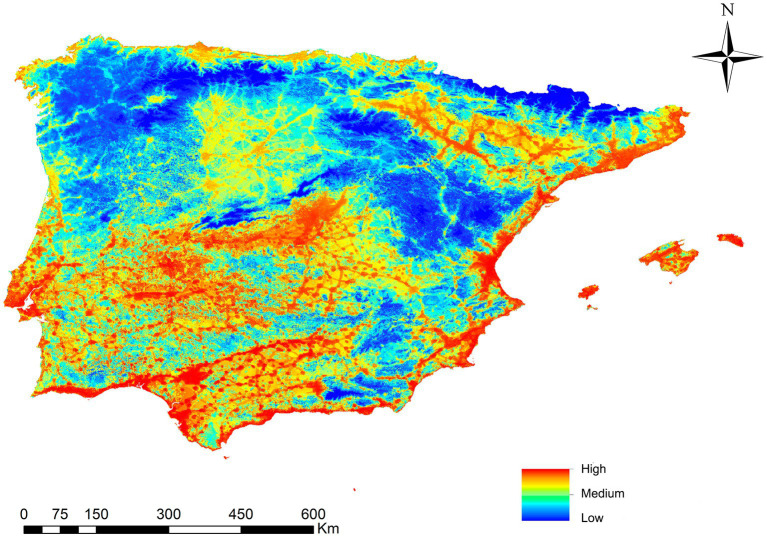
Ecological niche model of the risk of *Leishmania infantum* infection in Iberian Peninsula (Portugal and Spain) and Balearic Islands (Spain).

Figure 4 presents the map with the potential risk of transmission by *L. infantum* in the mainland and the Balearic Islands. Different infection risk values are represented using a color palette from red to blue, with the highest value being 0.56 and the lowest 0. The territory is divided into five risk ranges established by natural jenks (Very High, High, Medium, Low and Very Low), with 23.8% of the study area identified as very high/high risk areas, 23% as medium risk areas, and 53.2% as low/very low risk areas. There is a risk of infection throughout the study area except for high-altitude areas. The places with the highest risk of transmission correspond to the southwest and center of the peninsula, as well as the coast near the Mediterranean Sea, the Balearic Islands and the Ebro basin, places which coincide with areas characterized by ideal *Ph. perniciosus* habitats and high infection rate. Areas such as the northern plateau and the north and northwest coast have intermediate risk values. Areas of the interior of the peninsula, mountainous areas and higher altitudes with cooler temperatures exhibit risk values close to zero.

### 3.4 Validation of the *Leishmania infantum* potential transmission risk map

**Figure 5:**
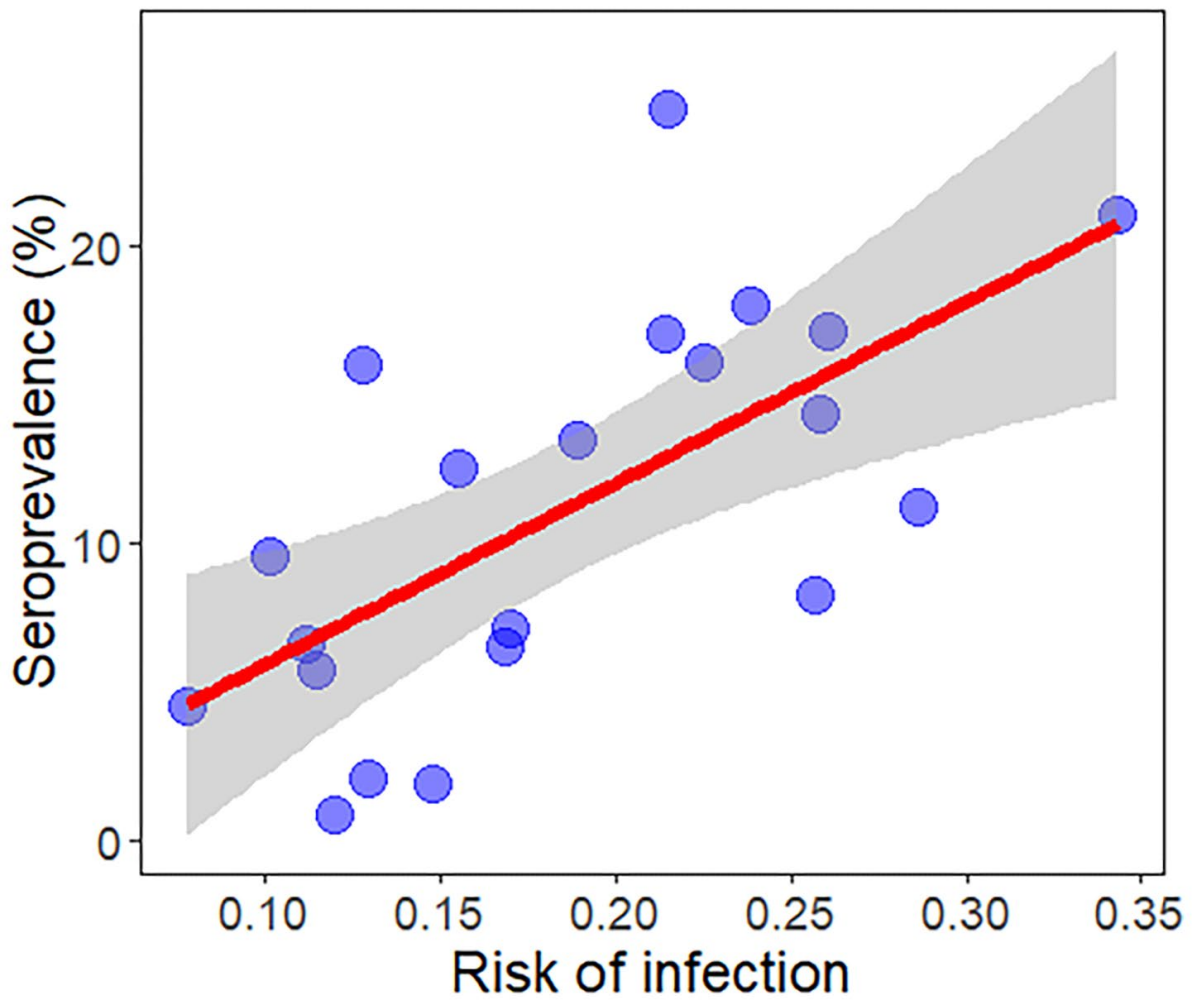
Regression plot for the validation of the ecological niche model between the mean risk of infection and disease prevalence in dogs most recently to date in all Spanish autonomous communities and Portuguese regions of the Iberian Peninsula and in the Balearic Islands (Spain) reported by Almeida et al. (13) and Montoya-Alonso et al. (16).

The result of the regression calculation was a positive and significant relationship between the infection risk map for *L. infantum* and the seroprevalence in infected dogs by each autonomous community in Spain and regions in Portugal (β ± SE = 61.15 ± 16.39, *R*^2^ = 0.42, *p* < 0.01) (Figure 5). The results obtained from the unweighted vector ENM did not fit significantly with the seroprevalence data (β ± SE = 20.79 ± 12.38, *R*^2^ = 0.13, *p* > 0.05), highlighting the importance of combining it with the infection rate.

**Figure 6:**
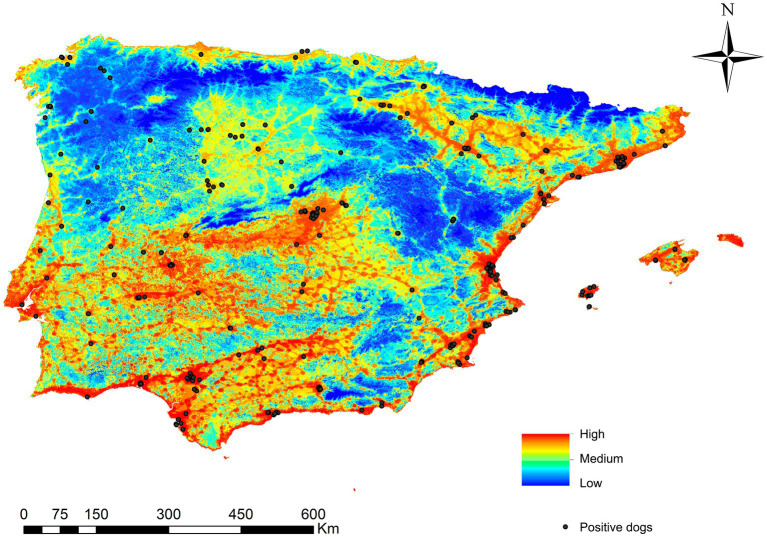
Ecological niche model of the risk of *Leishmania infantum* infection in Iberian Peninsula (Portugal and Spain) and Balearic Islands (Spain) and the geolocations of infected dogs in Iberian Peninsula and Balearic Islands (Spain).

Regarding the dogs infected with *L. infantum* and geolocated, 82.6% were in areas estimated to be at very high/high risk areas, 13.2% in medium risk areas and 4.2% in low/very low risk areas (Figure 6).

### 3.5 Forward projection of potential risk of transmission de *Leishmania infantum*

**Figure 7:**
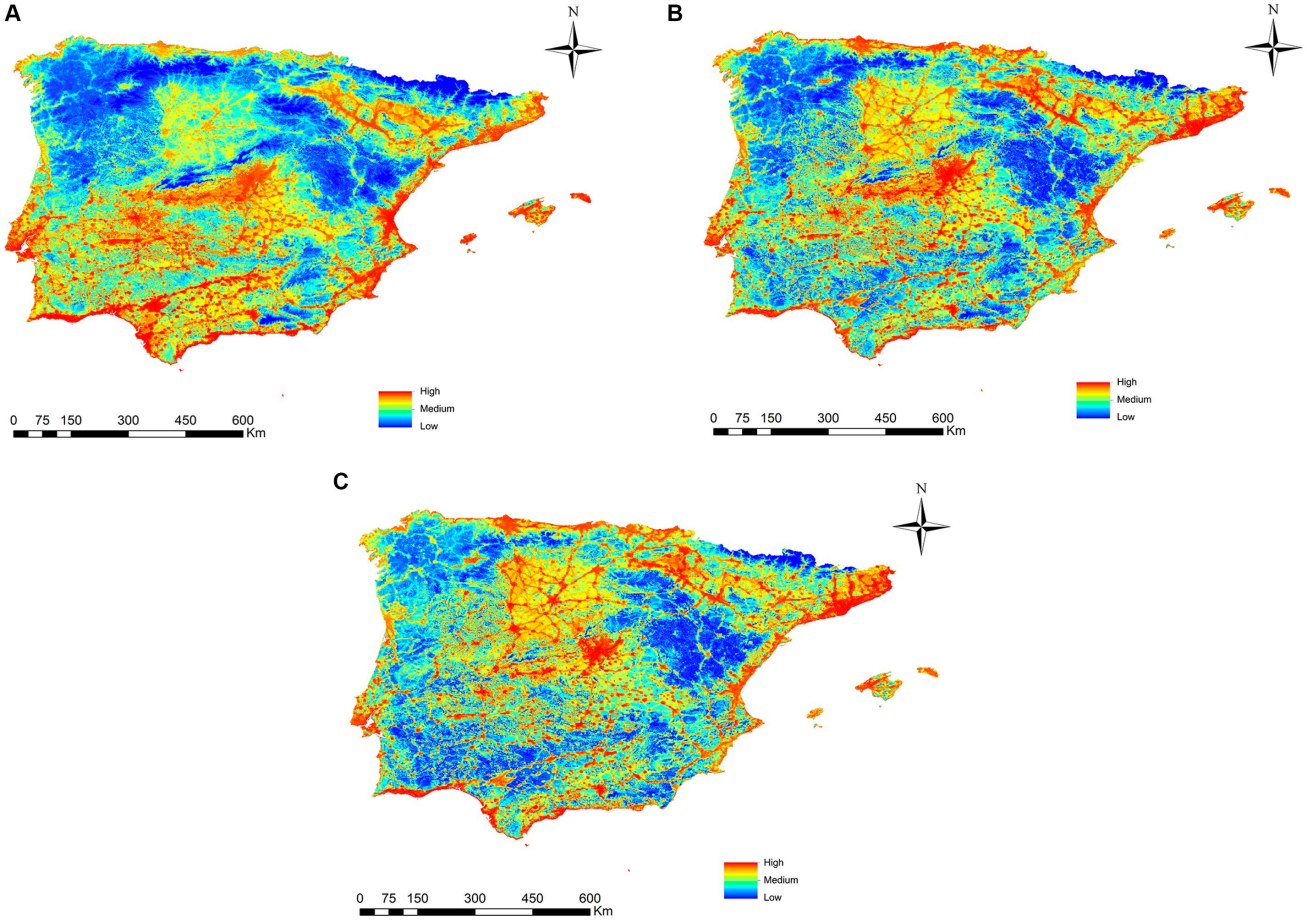
Projections of the risk of *Phlebotomus perniciosus* infection for 2040 **(A)**, 2060 **(B)** and 2080 **(C)** in Iberian Peninsula (Portugal and Spain) and Balearic Islands (Spain) under the climate change scenario RCP 8.5.

In the projection of potential transmission risk maps to the three future scenarios (2040, 2060 and 2080) of *L. infantum* through *Ph. perniciosus*, a latitudinal shift of the risk of infection toward the north of the peninsula is observed in both 2060 and 2080 (Figure 7). When the range-change analysis was carried out, the percentage of the territory where the risk of infection for *L. infantum* increases was in the North of the peninsula with 4.5% by 2040, 71.6% in 2060 and 63% in 2080. However, there is also a loss in the percentage of territory where there is a risk of infection, mainly in the south of the peninsula, being 9.6, 14.4 and 27.9% for 2040, 2060 and 2080, respectively.

## 4 Discussion

This study provides quantitative data on the risk of *L. infantum* infection in Iberian Peninsula (Spain and Portugal) and the Balearic Islands (Spain). The novelty of this study lies in the weighted use of both the calculation of habitat suitability by ENM of *Ph. perniciosus*, and the infection rate of *L. infantum* in the sandfly to predict the presence of the vector and the infectivity of the disease more accurately.

Prior to our study, the methodology of the ENMs has already been applied to try to model the distribution of leishmaniosis both in Europe and in other continents, using data on either the presence of vectors or infected hosts (17, 24–32). The same is applied to the Iberian Peninsula, where there are only two GIS studies have individually use the records of infected hosts or the distribution of their vectors, respectively (33, 34).

The risk map proposed in this work combines the potential distribution of the main vector of *L. infantum* in the Iberian Peninsula and the calculation of the parasite infection rate in the vector to model the risk of contracting the disease in a more realistic way. In fact, this weighting strategy improves the predictive power of the resulting model (*R*^2^ = 0.42, *p* = < 0.01) compared to the *Ph. perniciosus* suitability model alone (*R*^2^ = 0.13, *p* = > 0.05).

The variables that contribute most to explaining the potential distribution of *Ph. perniciosus* are the human footprint (built environment, population density, electric power infrastructure, cropland, grazing land, roads, railways, and waterways) and BIO_1_ (Mean Annual Temperature). Areas where human pressure is high are an ideal habitat for the maintenance of *Ph. perniciosus* populations. These areas with high anthropic presence, such as parks and agricultural land, also have important reservoirs of *L. infantum* (rabbits, rats, cats) associated with them, making it possible to efficiently maintain the biological cycle of canine leishmaniosis with high loads of infected sandflies (57–63). In addition, high prevalences of *L. infantum* infection in urban lagomorph populations have been linked to recent outbreaks of human leishmaniosis in Spain (57, 62), where annual incidences in humans (0.4–3.18 cases/100,000 inhabitants) and different prevalences in animals [29% in foxes (*Vulpes vulpes*), 13% in beech martens (*Martes foina*), 33% in wolves, 33.3% in rats, 15.6% in stray cats, 100% in rabbits, 8% in badgers and 1/3 of infected Egyptian mongooses] with special presence in southeastern Spain (11, 60–63) have been reported. On the other hand, the average annual temperature has a positive influence on the biology and ecology of the sandfly (rate of egg production, development of juvenile stages, annual number of generations, feeding behavior, period of activity and survival of adults) (64, 65). Other variables with a minor influence on the suitability models obtained include the diurnal mean range and the seasonality of rainfall. This last variable is also associated with the habitat types identified as influencing the distribution of hares and other wild reservoirs of *L. infantum* (natural grasslands, coniferous forests, lands occupied mainly by agriculture, lands with significant areas of natural vegetation and non-irrigated farmlands) that are characterized by moderate to high annual rainfall (66). Currently, areas with a higher seroprevalence of *L. infantum* in Spain suffer from drought, which may negatively influence sandfly populations and affect the transmission of the disease.

Regarding the variables associated with the rate of infection of *L. infantum*, the average annual temperature also influences its development, with the percentage of infected sandflies increasing logarithmically as the temperature rises within their survival ranges (12).

Our combined risk model indicates that actually, areas of the interior of the peninsula, mountainous and higher altitude areas with low temperatures, (which decrease both the habitat suitability of the vector and the rate of infection of these by the parasite) have risk values close to 0. On the other hand, the areas with a higher risk of infection (the south-west and center of the Peninsula, as well as the coast near the Mediterranean Sea, the Balearic Islands and the Ebro basin) coincide with areas with a high human presence, high average annual temperatures and with the basins of large rivers such as the Tajo, the Ebro and the Guadalquivir.

In the case of future projections under climate change scenarios, an increase in the risk of infection by *L. infantum* can be observed in most of the territory (4.5% in 2040, 71.6% in 2060 and 63% in 2080), mainly in the northern part of the peninsula. However, in some areas of the south of the territory, there would be a decrease in risk over time (9.6% in 2040, 14.4% in 2060 and 27.9% in 2080), which may be due to the foreseeable decrease in water resources, and the reduction of wetlands and vegetation in these areas (67, 68). This work predicts that canine leishmaniosis, in line with other vector-borne diseases, will shift latitudinally and toward higher altitude areas, altering its dynamics both spatially and temporally, colonizing areas where it was previously absent (22, 32, 35, 36, 69, 70). The effect of climate change on the seasonality and distribution of these types of vector-borne diseases will be more pronounced within the temperature ranges conducive to transmission occurs (71, 72).

As future approaches to applying of ENMs in vector-borne zoonotic diseases, it is possible to use the weighting tool not only with the niche model of one of its vectors, but also with more than one that inhabit the same territory, each with different ecological niches, provides sufficient data are available to model their distribution. In this way, a more comprehensive model could be obtained to facilitate the prevention and control of these diseases by veterinary and other specialist personnel.

## Data availability statement

The raw data supporting the conclusions of this article will be made available by the authors, without undue reservation.

## Author contributions

IR-E: Formal analysis, Investigation, Methodology, Validation, Writing – original draft, Writing – review & editing, Data curation, Software. AB-d: Investigation, Methodology, Validation, Writing – original draft, Writing – review & editing, Data curation, Formal analysis, Software. MC-C: Formal analysis, Investigation, Methodology, Writing – review & editing. DB-B: Investigation, Writing – review & editing. SD-E: Investigation, Writing – review & editing. REH-L: Investigation, Supervision, Validation, Writing – review & editing. JÁS-A: Conceptualization, Investigation, Methodology, Supervision, Validation, Writing – review & editing. RM: Conceptualization, Funding acquisition, Investigation, Methodology, Project administration, Resources, Supervision, Validation, Visualization, Writing – original draft, Writing – review & editing.
