# Erratum: Analysis of the current risk of *Leishmania infantum* transmission for domestic dogs in Spain and Portugal and its future projection in climate change scenarios

**DOI:** 10.3389/fvets.2024.1436792

**Published:** 2024-06-04

**Authors:** Frontiers Production Office

**Affiliations:** Frontiers Media SA, Lausanne, Switzerland

**Keywords:** *Leishmania infantum*, leishmaniosis, *Phlebotomus perniciosus*, Spain, Portugal, dogs, ecological niche model, infection risk

Due to a production error, there was a mistake in the order of Figures 5, 6, which were swapped incorrectly while the order of their captions remained unchanged.

Figures 5, 6 and their correct legends appear below:

**Figure 5:**
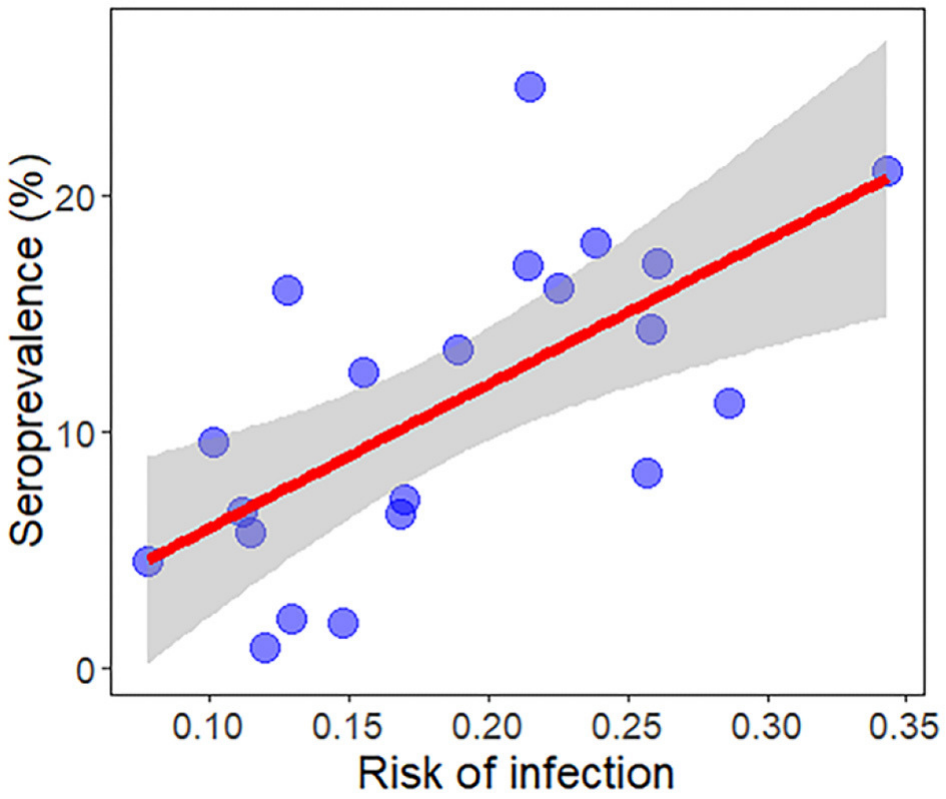
Regression plot for the validation of the ecological niche model between the mean risk of infection and disease prevalence in dogs most recently to date in all Spanish autonomous communities and Portuguese regions of the Iberian Peninsula and in the Balearic Islands (Spain) reported by Almeida et al. (13) and Montoya-Alonso et al. (16).

**Figure 6:**
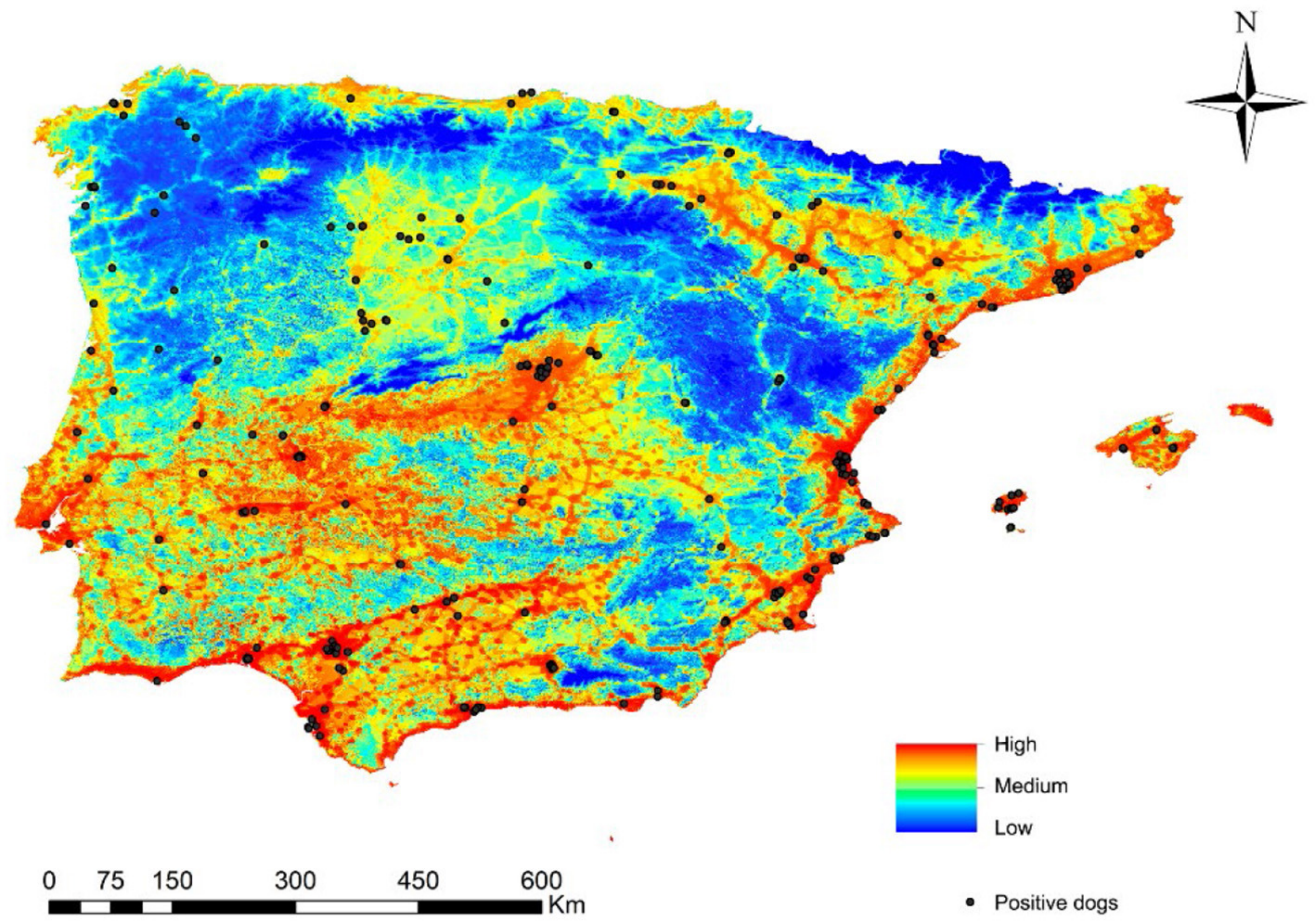
Ecological niche model of the risk of *Leishmania infantum* infection in the Iberian Peninsula (Portugal and Spain) and Balearic Islands (Spain) and the geolocations of infected dogs in the Iberian Peninsula and the Balearic Islands (Spain).

The publisher apologizes for this mistake. The original article has been updated.

